# Women’s well-being and reproductive health in Indian mining community: need for empowerment

**DOI:** 10.1186/1742-4755-10-24

**Published:** 2013-04-19

**Authors:** Melba Sheila D’Souza, Subrahmanya Nairy Karkada, Ganesha Somayaji, Ramesh Venkatesaperumal

**Affiliations:** 1 Department of Adult Health and Critical Care, PO 66, College of Nursing, Sultan Qaboos University, Al-Khoud, Muscat 123, Sultanate of Oman; 2 Department of Business Studies, Higher College of Technology, Al Khuwair, Muscat, Sultanate of Oman; 3 Department of Sociology, Goa University, Taleigao Platuea, India

**Keywords:** Well-being, Reproductive health status, Gender preference, Domestic violence, Marital relationship, Qualitative design, Nursing

## Abstract

This paper is a qualitative study of women’s well-being and reproductive health status among married women in mining communities in India. An exploratory qualitative research design was conducted using purposive sampling among 40 selected married women in a rural Indian mining community. Ethical permission was obtained from Goa University. A semi-structured indepth interview guide was used to gather women’s experiences and perceptions regarding well-being and reproductive health in 2010. These interviews were audiotaped, transcribed, verified, coded and then analyzed using qualitative content analysis. Early marriage, increased fertility, less birth intervals, son preference and lack of decision-making regarding reproductive health choices were found to affect women’s reproductive health. Domestic violence, gender preference, husbands drinking behaviors, and low spousal communication were common experiences considered by women as factors leading to poor quality of marital relationship. Four main themes in confronting women’s well-being are poor literacy and mobility, low employment and income generating opportunities, poor reproductive health choices and preferences and poor quality of martial relationships and communication. These determinants of physical, psychological and cultural well-being should be an essential part of nursing assessment in the primary care settings for informed actions. Nursing interventions should be directed towards participatory approach, informed decision making and empowering women towards better health and well-being in the mining community.

## Background

In India, the reproductive years for women are of central importance to their lives. Women’s role in reproductive health (RH) is affected by, and could influence her status and empowerment as an individual [1]. The choice of livelihood, where to live, who to marry, number of children, freedom of movement and choice of friends are critical decisions for women which may empower them to become matriarchs of the family. Empowerment is defined as the expansion in women’s ability and freedom to make these strategic life choices; a process that occurs over time and involves women as agents who have the ability to formulate choices, control resources, and take decisions affecting important life outcomes [2-5]. Reproductive health (RH) and empowerment expressions for Indian women are often misunderstood in the context of the Indian health care system. A number of studies have shown that women may be empowered in one area of life while not in others [2,5,6]. Gender influences and differences characterized by patrilineal descent, patrilocal residence, inheritance, and succession practices exclude women in India [7]. In a survey of 536 women in India, findings revealed that empowerment was viewed as having control over enterprise income and having a decision-making ability over the household [8]. However, particularly in the rural communities, Indian women have a subordinate position compare to men and have less power, autonomy status and independence.

Mining intensive operations and skilled activities also tend to reduce opportunities for women in Orrisa, India; thus increasing their levels of dependency and vulnerability [9]. In the coal mines of Orrisa women face discrimination and have limited choice regarding their health, safety and security [10,11]. Nearly every woman experiences one or two miscarriages/ stillbirths/ foetal defects and congenital disorders in these areas. Moreover, women face abuse by alcoholic husbands , partner infidelity, and risk of Human immunodeficiency virus / acquired immunodeficiency syndrome (HIV/AIDS) and other sexually transmitted diseases (STDs) as a result. Workplace discrimination in local communities also tend to disempower women [9]. In a study among 145 Indian women living in mining areas, women reported a loss of negotiating power, and poor health and well-being. In a study among 145 women in a mining community women had less decision making and reproductive health choices [12]. Spouses rather than the women themselves made reproductive choices about avoiding conception (40%), spacing (39%), number of children (39.3%), use of contraceptives (35.8%) and permanent family planning (33.1%) compared to women in the mining community [11]. Predictors of physical and mental health scores were illness, domestic violence, employment, social and cognitive coping [12]. Few attempts have been made to investigate the perceptions of Indian women living in mine communities about their well-being linked to RH.

This paper is an exploratory qualitative approach designed to examine the cumulative influence of structural (education, employment, age, family arrangement) personal (RH choices), cultural (son preference), and relational (marital relationship, domestic violence) on well-being and RH. Examining these diverse influences can better inform programs and policies to support and empower women and to improve their well being and RH in the mining communities.

### Conceptual framework

**Figure 1:**
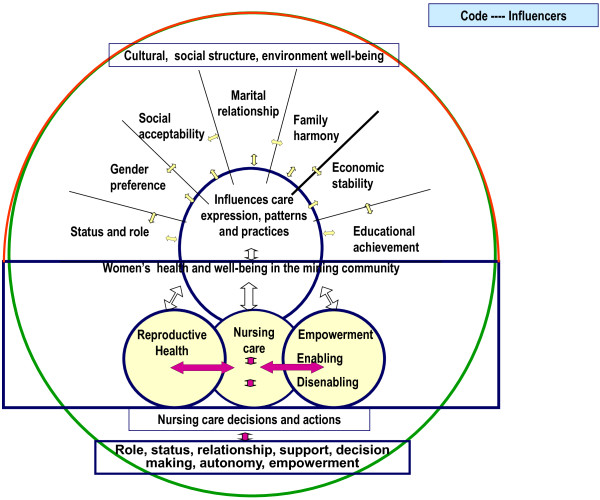
Leninger’s sunrise model for women’s well-being and reproductive health in the mining villages.

The theory of culture care diversity and universality or Sunrise model (Figure 1) was used to explore the well-being and RH among women in the domain of inquiry [13,14]. Within this conceptualization, RH and care is seen as a major enabling (e.g. mobility) or disabling (e.g. male dominance) factor linked to empowerment. This model shows how cultural, social structure, environment well-being influence care expression, patterns and practices among women. In this study, the model was used to explore diverse and universal care values (physical, psychosocial, emotional, spiritual, mental, health and well-being) from the emic (woman’s) perspective in local mining community. The model shows the relationships between the totality of influences on structural, personal, cultural and relational factors within a woman’s world. Women in the mining community are caring and capable human beings concerned about their immediate family. The dynamic environment surrounding the women is closely related to the concept of culture and care. Health includes RH status, wellness and roles that reflects the ability of the women to perform their daily activities reflected by cultural values, beliefs and convictions. Nursing uses various modes of action (e.g. acceptability, preservation, accommodation, negotiation) suited to the women’s well-being. This model focuses on the patterns and expressions linked to women’s perceptions and meaning of well-being and RH. Socio-economic, cultural, education, family, personal self, mental well-being and other factors impinge on women’s RH. Nursing care decisions and actions should be based on women’s role, status, relationship, support, decision-making, autonomy and empowerment in the mining community.

### Aim

This paper is a qualitative study to explore the perspectives of and factors influencing the well-being and RH among women in the mining communities in India.

## Methods

### Design

The purpose of this study was to describe perspectives of and contextual meanings of well-being and RH dimensions of women living in the rural mining region of Orissa from an “emic,” or insider’s, perspective. For this study, an exploratory qualitative design was used and the conventional qualitative content analysis approach undertaken [15].

### Setting

The selected mining community lies in the iron ore mining belt of Goa with a population of 40,000; of which 40% are dependent on mining and 25% on agriculture for the past fifty years. The Goan economy is heavily dependent on the iron ore industry and allied activities, forms a major share of the regional income and contributes to the state gross domestic product. Goan iron ore forms the bulk (60%) of iron ore exports in India. Mining activities have had an impact on the natural environment (air, surface and groundwater and the land adjacent to the mine sites) and have positive and negative impacts on the health and well-being of the people living in these mining communities [16]. The percentage of women reporting health problems are higher (91%) than the percentage of men (85%) reporting these problems [17]. Women are not employed in the iron ore mining belt in Goa due to the nature and intensity of work.

### Sampling and sample

A purposive sampling was used to select women living in the mining communities in Goa and who reported experiences which negatively impacted their RH choices such as gender preference, wife beating or inebriated husbands. Forty women were screened by the investigators for the inclusion criteria and findings of the previous study [11]. The selected women were recruited after obtaining voluntary consent and willingness to participate in the study. The spouse of the selected women were informed of the study but were not included in the study.

### Data collection instrument

A semi-structured in-depth individual interview guide (Appendix A) and baseline survey were used to meet the study objectives. This guide was prepared based on an extensive review of the research and lay literature related to RH, family support, work, education, health seeking behaviours, empowerment and social life of women living in the mining community in India. Focus group discussions were held with women whose spouse worked in mining communities, teachers, doctors, and women in local authority body. Academic expert opinions and suggestions were also taken into consideration in determining the important areas to be included in the interviews. The English version of the guide was validated by four experts (psychologist, sociologist, psychiatrist and physician) and was found to be valid. The tool was then translated to the local language (Konkani) by a language expert or linguist. The investigators conducted the individual interviews.

### Ethical approval

The research study conformed to the ethical guidelines of the Indian Council of Medical Research [18]. This study was approved by the Research and Ethical committee of the Goa University. Written and oral information regarding the study was given to the women selected through purposive sampling. These women were informed about voluntary participation and their right to withdraw at any time without detriment. They were assured of anonymity and confidentiality during data coding, validation of results and use of aggregated data for dissemination. Written informed consent was obtained from all the selected women.

### Validity and rigour

The interview process was carefully planned in advance to elicit trustworthiness, credibility, transferability and auditability in the qualitative inquiry. Credibility criteria involves establishing the results of a qualitative research that are believable to the participants of the study [19]. Credibility was strengthened by having 10 women participants validate interpretation of the findings and verify the accuracy of the interview transcripts that reflected their actual experiences. The findings of this study were also shown to all the researchers who were able to recognize the experiences of the women based on their past experiences and discussions with key informants in the local villages. Transferability, or fit, was established through “thick description” [20]. The researchers collected a detailed description of data that included field notes together with a rich mix of participant quotations. Validity was established by confirming derived themes with women participants and the primary study interpreter. Reliability was established through consensual validation with an experienced qualitative researcher and auditability. Auditability was achieved when the researcher arrived at the same or comparable but not contradictory conclusions, given the researcher’s data, perspective, and situation [21]. The random transcriptions of the original study were given to the researchers for review who found similar categories and themes related to women’s disempowerment, including factors that affect the reproductive health phenomenon.

### Procedure for data collection

The investigators were familiar with the study objectives, methods, ethical protocols. They used a standardized protocol to communicate and interact, and build rapport with the women as they conducted the interviews and discussions. The investigators enrolled women who met the inclusion criteria and debriefed them about the nature and type of interviews. The investigators smoothly interacted with women and informed them regarding the study protocol and written consent. To promote woman’s dignity and respect due to the sensitivity of the data, these interviews were carried out in privacy of their homes. Confidentiality was maintained throughout the study. The semi-structured interview guide was used by the investigator to interview the selected women during June-August 2010. The interviews were conducted for 90–120 minutes until data saturation. The responses were tape recorded and the oral interviews were recorded on mini-discs for transcription. These interviews were transcribed and translated by bilingual researchers. Interview tapes and questionnaires were stored securely.

### Method of analysis

Thematic content analysis and constant comparative techniques allows investigators to make inferences from text or other media that are valid and replicable. Procedures used in content analysis vary depending on the purpose of its use [22,23]. Clear steps for conducting the qualitative content analysis were developed based on the work of experts to ensure that every investigator was using the same analysis technique [24]. Steps included reading through the transcripts several times, identifying significant statements (i.e., meaning units), clustering these into subcategories and categories, and finally identifying underlining threads or themes. The reliability was established by percent agreement using key words, statements, most emphasized, number of repetitions and reinforcement. The intercoder reliability was done using double data entry and double coding. Inter-rater reliability was protected by independent analysis by each investigator who read through the responses a minimum of three times to become immersed in the data.

Open coding was used to identify patterns of responses, selectively coded and transcribed. The codes were grouped together in conceptual categories that shared similar patterns or related content; then the conceptual categories were inductively abstracted into larger conceptual entities labeled as themes. A thematic content analysis and tabular grid was used for cross-referencing and examining concepts and inter-relationships between empowerment and reproductive health were examined. The data were then dissected and categories of relevant relationships and processes identified for later data analysis. Within each category, relationships and processes were uncovered for each woman interviewed. Threats to interpretative validity were diminished by literature review and data analysis by all researchers. There were sufficient and repeated themes to validate category formation, until data saturation was achieved. After core categories were identified through selective coding, a matrix of theoretical concepts was completed. Consequently, these theoretical concepts were used to create a more detailed matrix of the RH linked to disempowerment phenomena under study. The themes were abstracted into larger, more conceptual patterns of meaning that helped us understand how the women experienced reproductive health and empowerment. The categories were then searched for patterns and insights regarding dimensions of empowerment and with a particular focus on well-being and reproductive health [25].

## Results

### Socio-demographic characteristics

Most of the married women in the mining community were above 30 years (60%) while 27.5% of the women were separated/ divorced (Table 1). In total, 50% of the women reported having no education and half were unemployed (47.5%). Some of the women had difficulty meeting the family needs (47.5%) and slightly over half (52.5%) reported poor health.

**Table 1:** Socio-economic status among women in the mining community N = 40

**Socio-Economic Status**		**Percentage**	**Frequency**
Age	Less than 30 years	40.0	16
	31 - 50 years	60.0	24
Marital status	Living with husband	72.5	29
	Separated / divorced	27.5	11
Educational status	No schooling	50.0	20
	Middle school (up to 7 grade)	35.0	14
	Higher secondary (up to 12 grade)	15.0	6
Gainful employment	Employed for cash	30.0	12
	Employed for kind	22.5	9
	Not employed	47.5	19
Ability to meet family expenditures	Manageable	20.0	8
	With difficulty	47.5	19
	Poverty	32.5	13
General health status	Excellent	15.0	6
	Good	32.5	13
	Poor	52.5	21

### RH characteristics

The majority of women (65%) in general were married at ages of less than 18 years (Table 2). Slightly more than half (57.5%) had more than four children, with less than 2 year birth intervals (52.5%). The main method of contraception was tubal ligation/ tubectomy (13%) and contraceptive pills (9%). Spouses made most of the RH choices like spacing/birth intervals (82.5%) and son preference (95%) and over two thirds (67.5%) of the spouses drank alcohol heavily husbands took alcohol. The majority of the women reported wife beating or domestic violence (90%) in various forms. Only about one in five (22.5%) of the women reported good inter-spousal communication. Only a few women were able to make personal, family or household decisions like moving freely in the community (27.5%).

**Table 2:** Reproductive Health Status among women in the mining community N = 40

**Reproductive Health Status**		**Percentage**	**Frequency**
Age at marriage	Less than 18 years	65.0	26
	More than 19 years	35.0	14
Number of children	Three and less	42.5	17
	Four and more	57.5	23
Birth intervals	2 years and less	52.5	21
	2 - 4 years	47.5	19
Methods used to prevent conception	Contraceptive pills	9.0	22.5
	Safe period/ Withdrawal	6.0	15.0
	Intrauterine device	4.0	10.0
	Condom	8.0	20.0
	Female tubectomy/tubal ligation	13.0	32.5
Reproductive health choices made by husband	Spacing/ birth intervals	82.5	33
	Number of children	72.5	29
	Son preference	95.0	38
	Use of contraceptives	47.5	19
	Abortion	40.0	16
Domestic violence	Any form of domestic violence	90.0	36
	Minor violence	55.0	22
	Major violence	35.0	14
Inter-spousal communication	Good	22.5	9
	Fair	45.0	18
	Poor	32.5	13
Husbands drinking habits	Daily alcohol intake	67.5	27
	Alcohol intake 2–4 times/ week	32.5	13
Decision-making	Household (e.g. cooking, child care)	40.0	16
	Family (e.g. assets, financial)	32.5	13
	Mobility (e.g. health care, work)	27.5	11

### Women’s well-being and reproductive health

Four major themes were identified from the interviews. These included socio-economic and environmental health well-being; women’s status, role and well-being; health, gender preference and cultural well-being and marital relationship, support and decision making (Table 3).

**Table 3:** Themes and categories from the interviews with women N = 40

**Themes**	**Categories**
Socio-economic, cultural and environmental well-being	Not having enough money
	Not having the basic necessities of life
	Less work opportunities
	Loss of agricultural fields
	Husbands are not financially support family
	No education for self and children
	Lack of clean water, sanitation, firewood, open spaces, forest produce
Women’s status, role and well-being	Gender differences/ inequality
	Performing socially constructed roles
	Less attention to self
	Inability to make reproductive health choices
	Feeling of powerlessness and isolation
	Less freedom of choice and mobility
Health, gender preference and cultural well-being	Susceptibility of acute and chronic illness
	Illness and dependence on medications
	Psychological stress and anxiety
	Gender preference
	Unable to obtain or use information
	Difficulties in obtaining help/ access to healthcare
Marital relationship, support and decision-making well-being	Wife beating
	Family disharmony/stress
	Poor marital relationship
	Strained relationship with in-laws
	No hope for the future

#### Theme 1. Socio-economic and environmental well-being

The main activities of women living in the mining communities were described as household tasks, child rearing, and in collecting forest produce and laboring in far off paddy fields and cashew plantations. There are no opportunities for women in the mining industry. Several women expressed lack of proper economic opportunities and unemployment or loss of income generating activities for family sustenance. Women expressed that mining has affected their work in the agricultural fields, leading to less productive opportunities. Woman 3: “I have poor financial condition as my husband is separated from me. I am tired, weak and unhappy about my present life due to isolation. I have to work hard to support my children, as we are poor and can't afford anything in life. I face poverty and health problems due to mining. Sometimes I depend on work in the mines otherwise we have finance problem.”

In many cases, the women reported that only a few fields were fertile or were cultivated due to the generation of huge amounts of mining dump materials flowing into nearby fields, ponds and streams (*nallah*) [26]. The impact of less agricultural fields, loss of income/economy and shift of occupation have exposed women to a different world at home and in the community. These impacts outweigh the positive factors of direct and indirect employment of their spouses in mining, small or medium business opportunities. Another woman (39-year): “My husband has no money. He does not look after me and my children. After the birth of my fourth daughter, he started ignoring me and left me at my mother's place four years ago. He tells me to look after myself and he never gives any money to me.” One woman described, “My children’s education is interrupted and my husband is not doing any work. It is difficult to live in this world as everything is expensive. I have no one to look after my five children. We have less food and live in poverty.”

As a result, most women did not feel accepted at home as they perceived themselves as poor achievers (poor education, unemployment, no income-generating activities, and non-productive activities). These women felt that they were unable to support the children and family and unable to meet the daily needs. Women are usually not included in decision-making and were socially and economically dependent on men to financially support them. For example, one woman (41-year) expressed feelings of uncertainty, fear and helplessness, “I am worried about my children and my future. I keep on thinking and I crying about the past and the unknown future. I wake up feeling uneasy, groggy and light headed. I have no one to tell my problems or share my unhappiness. I have to work hard until late night to support my children. If I am ill, I have no one to take care of me. My husband does not take me to the doctor. I feel hurt and I get thoughts of who will take care of my kids, in case I die. There’s no solution for it. If he (spouse) is good, then everything would be fine” (sic). Women are usually not included in decision making and are socially and economically dependent on men to financially support them. These findings impact emotional life and decision making in the family.

#### Theme 2. Women’s status, role and well-being

Some of the women have been displaced from their traditional settlements due to mining activities and were distraught over a lack of basic facilities such as clean water, sanitation, firewood and open spaces. They reported the erratic supply of water by water tankers of the mining companies and irregular bus transport. As the ground water has been affected by mining activities, this has led to dry wells and springs and little water for household use, shortages and erratic/irregular supply. As a result, as principal collectors of water, women had to collect water from far off sources. Thus, in the mining community, women spend a great deal of time and energy on providing for basic necessities of family and household needs like clean water for domestic use, food and fuel wood due to degradation of environment, less potable water and less opportunities of livelihood. These women expressed increased workloads with dual responsibility and household production.

Despite the fact that Goa has an uniform civil code for gender equality in law that potentially enables women to have a greater say in what happens to land, participants expressed gender inequality from certain provisions within the law which reduced their decision-making powers. Land is important to women as a result of greater dependence and involvement in the agricultural fields and opportunities of diversified livelihoods, as men worked in the mines or other jobs. The ownership of productive assets, such as arable land, is crucially linked to viable livelihoods, to a lower risk of poverty, to access to credit, and to a greater ability to bargain for higher wages. Woman 4: “It is not easy to live with my husband, he is rough and tough. I have to listen to him. He took the compensation money for our land, but did not give any money to us. He had changed a lot since marriage and I do not know how much he earns. I never had a happy marriage and I did not get much affection. My husband never gives me or my children any money. I live in my parent’s home and my mother supports me.”

As the women revealed, several of the spouses received small one time compensation payments for land lost to mining activities. This money was used by the spouse to drink, smoke, gamble and other self-gratification needs leading to impoverishment of the family. Other spouses worked in mining operations and driving heavy vehicles, resulting in excessive work tension and the need to put in long tedious hours. The women reported that this resulted in the emergence of social vices by the husband such as alcoholism, wife harassment and negligence of family. In addition reduced leisure space, strong peer influence and stressful jobs lead to less family commitments and more time spent outside homes. Woman 3: “My husband beats me when he drinks without any reason. Many times he beats me, fights and abuses me with filthy words. He does not provide financial support and has no job. If he is drunk he harasses me a lot and sometimes he demands money which is beyond my means. He comes home very late, he listens to his family and never to me”.

Women indicated that physical abuse was not as frequent as verbal and emotional abuse but that it was more intense, caused psychological trauma, and affected their self-concept, especially if it occurred in front of the children: “He drinks alcohol and comes home late, he listens to his family and never to me. He gets angry, scolds (abuses, bad words), shouts, fights and starts abusing till his anger or frustration is reduced or he is tried. Sometimes he beats me but not always and the children are afraid of him. He does what he likes, doesn't listen to any of his family members” (sic). Women described the stigma of divorce and separation from the children. Women explained that they were afraid to end this kind of life, and if they were divorced, they would be denied their children or a normal status in society.

#### Theme 3. Health, gender preference and cultural well-being

Women expressed increased susceptibility to illness/diseases (e.g. skin, respiratory diseases like tuberculosis, gastro-intestinal) among children and family members and attributed them to changes in the quality of air and water, degradation of soil, mining accidents etc. Moreover, women reported increased illness such as colds, headaches, fever, dry cough, eye allergy, dust allergy and throat irritation in a mining village. One women shared her experiences as follows: ‘I just feel like ending my life. The main tension is because of the unemployment of my husband. Since it’s summer now, he can go anywhere (and do odd jobs), but once it starts raining, where can he get a job. My elder son has jaundice. If one of them is better, then the other one falls ill. I’m worried because we have to buy the medicine prescribed by the doctor. It depends upon my husband’s work and his moods because his moods change frequently. My husband has lots of debts. I have sleepless nights. I often get headaches, body aches, abdominal pains and giddiness when I think about all this. I have my own tensions with my family and my husband. I am fed up with everything and the monotonous life. I am not feeling well. My back is pain and general health is low.” (sic)

Women expressed inability to make personal, family or household decisions due to spouse dominance, superiority and power exerted to control wife’s mobility, behaviors and RH choices. Women also expressed fear, anxiety, depression and nervousness of becoming a mother due to the pressure of a son and non-acceptance of a girl child. They experienced strained relationship and stress with the husband and mother-in-law. One woman remarked about the unhappiness expressed by her in-laws after giving birth to a girl child. “But mother-in-law is not happy with me and baby, as they wanted a boy baby so they harassed, insulted, scolded and hurt me. They were unhappy with my three daughters and did not treat us well.” Another woman: “I felt bad because everyone was expecting a son and since this did not happen I was thinking, ‘what will my people say! I was afraid of my in-laws. It makes no difference to me because, after all, it’s God’s will. I am only afraid of them (in-laws), of what they will say or what will they do to me. I am really worried, sad and depressed”.

Women also described the control of the husband over their lives and in-laws who shared in, controlled, and interfered with the major private family decisions like male child preference. “Because I gave birth to three girls, my mother-in-law was very unhappy and fought with me every day. Also, whenever I had arguments with others in the village, they ridicule me for not being able to bear a son. I could not keep my head up, I felt awful and a lot of pressure on me” (sic). (A 40-year-old woman who had 12, 10 and 8 year-old daughters).

#### Theme 4. Marital relationship, support and autonomy

The women described their suffering, physical, emotional and verbal abuse by their husbands in terms of forms, intensity, and psychological consequences of the abuse. Women remarked that spouse harassment, assault, abuse, rigidness, dominance and unsupportive nature led to marital disharmony. Sometimes their spouses did not provide financial support due to unemployment or drinking habits. The women indicated that physical abuse was not as frequent as verbal and emotional abuse but that it was more intense, causing psychological trauma, and affected their self-concept and self-esteem, especially in the presence of their children: “He drinks alcohol and comes home late, he listens to his family and never to me. He gets angry, scolds (abuses, bad words), shouts, fights and starts abusing till his anger or frustration is reduced or he is tried. Sometimes he beats me but not always and the children are afraid of him. He does what he likes, doesn't listen to any of his family members” (sic).

Other cultural rules mentioned by these women were the stigma of divorce and separation from the children. Women explained that they were afraid to end this kind of life, and if they were divorced, they would be denied their children or a normal status in society. The women described psychological and emotional consequences, low self-esteem and social isolation due to different forms of abuse. The women were able to describe their “deep-down” feelings in detail, and with courage, they described the degree of their feelings of helplessness and hopelessness and the stage of their despair. Feelings of helplessness made some of the women unable to execute their roles as wife and mother. They expressed feelings of separation and isolation from spouse. Women 5: “I cry the whole day and I have heaviness in my head, my head throbs and I have severe migraine with my marital relationships. It is not worth living like this. My husband has no feelings or understanding. He is wild and abusive. He likes to wander around leaving the children and me at home. I have lots of “tension” in the past 2 years due to constant verbal and physical fights with him. Every time my mother-in-law would look at me and say bad words to me. Because of all this, I have lots of tension and could not get sleep.” (48-year woman malnourished and sick).

The women described psychological and emotional consequences, low self-esteem and social isolation due to different forms of abuse. The women were able to describe their “deep-down” feelings in detail, and with courage, they described the degree of their feelings of helplessness and hopelessness and the stage of their despair. Feelings of helplessness made some of the women unable to execute their roles as wife and mother. Woman 1: ‘When I think too much, I become stressed, have no appetite and so I can’t eat or do anything for that matter. I feel I don’t need anybody. I just feel like ending my life. The main tension is because of the unemployment of my husband. Since it’s summer now, he can go anywhere (and do odd jobs), but once it starts raining, where can he get a job. My elder son has jaundice. If one of them is better, then the other one falls ill. I’m worried because we have to buy the medicine prescribed by the doctor. It depends upon my husband’s work and his moods because his moods change frequently. My husband has lots of debts. I have sleepless nights. I often get headaches, body aches, abdominal pains and giddiness when I think about all this. I have my own tensions with my family and my husband. I am fed up with everything and the monotonous life. I am not feeling well. My back is pain and general health is low.” (sic)

## Discussion

The findings of the study show that married women adopt the role of a wife, mother and care giver as a primary identity, even when they work outside the home. These findings relate to Indian social and cultural rules that reflect male dominance, superiority over women, and their right to control and correct a woman if she thinks, feels, acts or does something unapproved or if she does not obey her husband. Women’s perceptions of low education, low social status, economic instability, gender inequality and lack of decision making were disabling factors of empowering in the mining community. Women who earn substantially very little have no influence on their husbands economic and social activities [27]. The acquisition of private lands for mining and destruction of several forests led to women losing access to land based work opportunities with no alternative sources of income. They lost their economic and social status, and were not able to participate in agricultural activities and processing of forest products [28]. Higher age, family economic status, fewer number of children, absence of domestic violence, absence of reproductive and enduring illness are important determinants of quality of life and reproductive health indices [11]. Early marriage, and yearning for a male child results in frequent child bearing during the reproductive years [29]. Birth intervals of 3–5 years are healthiest for mothers and their babies [30]. Son preference is a strong predictor of short birth intervals [2]. Hence the extent of control over women and their reproductive choices is determined by social-structural (like economic ability), health, gender factors and resources affecting sharing of power, resources, and social positions.

Husband’s took decisions regarding where and when women ought to seek reproductive choices and access to health care. The gender distribution of roles or division of labour (central role in reproducing the social order) and power relations have reduced decision- making among women and have forced them into positions of subordination. Boy preference was most marked for mothers who had girls and was often expressed through lack of support and hostility from the husband and mother-in-law. One-third of spouses took independent decisions regarding RH choices like avoiding conception, spacing, number of children, use of contraceptives and permanent family planning [11]. Early marriage among girls may lead to powerlessness and difficulty in expressing reproductive choices like negotiating contraception use [31]. Marriage and family laws often prescribe a younger age of marriage for women than for men and restrict women to childbearing and service roles, while denying them equal opportunities available to men [32,33]. Lower age at marriage has been associated with decreased autonomy through its positively association with education, media exposure, and premarital employment [34].

Women with lower perception of environmental mastery and self-acceptance were subject to higher rates of abuse in the mining community. These women who feel disempowered are more likely to be abused. But women who have a sense of mastery and competence in managing the environment, who can control a complex array of external activities and make effective use of surrounding opportunities, and who have a positive attitude toward themselves are subject to lower rates of all forms of marital abuse. Alcoholism among spouses led to cognitive imbalance among husband’s and conflict among spouses. This is an important risk factor for emotional disturbance among women and family disharmony leading to broken families. Women partnered to miners were at double the risk of social-psychological abuse. Women partnered to men engaged in some form of shift work were more than four times as likely to have experienced recent physical abuse [35]. A home environment where women are beaten is less likely to promote women empowerment or autonomy [2]. In Goa, 40% of women reported reproductive health problem, violence and abuse, poor mental health and other risk behaviours [36]. 35% of Indian women of reproductive age reported having experienced physical domestic violence at some point in their married lives [37,38]. Younger age, higher economic status, absence of domestic violence and enduring illness, and better cognitive and behavioural coping among married women (N=145) significantly predicted better SF36 scores. The combined effect of predictors on physical and mental health show illness, domestic violence, employment, social and cognitive coping as significant predictors among women in the mining communities [12].

More than three-fourth of the adult women experience violence at the hands of their husbands at some point during their lives. The prevalence of physical domestic violence perpetrated by husbands, is staggeringly high across the Indian subcontinent. The effect of abuse on women’s self-esteem is devastating and increases the risk for mental health problems [39]. Women abused reported higher than normal rates of depression, helplessness, and hopelessness [40]. In recent studies, from various parts of the world, women reported being hit or otherwise physically assaulted by an intimate male partner at some point in their lives [41] and reported exposure to different types of physical violence [15]. Men spent more time drinking with their work mates and less time at home with their families or in productive leisure pursuits due to traditional views on masculinity and gender roles. Women felt powerless in their marital relationships due to structures of male power that ended in arguments and negative outcomes [42].

In this study disempowerment among women is crucially linked to the quality of her relationship with her husband or children. Social status, economic costs, occupation, age of marriage, and residence account for fertility decline among women [12]. There is an inverse relationship between women's RH and their education, employment and economic status [43]). Among 14,779 ever married women aged 15–49 years significant factors (1995–96) like participation in health care, work exposure, family structure, equality in marriage, and marital status variables were found to influence women’s empowerment [2]. The ability to control woman’s well-being is an important precondition for improving reproductive health among women. The likelihood of spousal communication about family planning and contraceptive use is significantly higher among married women who exercised control over choice regarding husband and marriage than among those who have arranged marriage [33]. This study suggests socio-economic, health and psychosocial antecedents and resources as factors of well-being and reproductive health leading to disempowerment. The quality of marital and intimate relationships is a significant factor in determining a person’s psychological well-being. The psychological characteristics of work (relations with colleagues, decision-making, role overload, insecurity) structural aspects of work (work hours, shift patterns, weekend work) and patriarchal culture in the mining community generate work-family conflict impacting on the well-being of workers and their family members [44]. In the Sunrise model, a combination of cultural expectation of ‘male preference’ and husbands alcoholism resulted in a vicious cycle of family disruptions, poverty and domestic violence. Four main themes in confronting women’s well-being are poor literacy and mobility, low employment and income generating opportunities, poor reproductive health choices and preferences and poor quality of martial relationships and communication. The study provides insight into the role that primary care or community health nurses could play in enhancing care among rural women regarding health education, counseling, screening, cognitive behavioural change as well as encouragement to seek health care for reproductive health issues. The influence of boy preference, much reported in South Asia and found to be a major effect modifier [45]. A key component of women’s power is their access to and control over economic, political, and social resources, including employment [4,46]. Empowerment is influenced by reproductive events, such as the birth of a baby and especially, a son and life-stage indicators like age, education, employment, power, economic status [2].

Empowering women through education, counseling, employment, and eventually helping women achieve equal opportunity in getting a job and finance themselves without the need for being dependent on their husbands is another important issue to be considered. Empowering women in these rural mining communities through regular home visits and counseling by nurses; using community leaders, local *panchayats*, and women’s association in collaborative activities; establishing empowerment programs that directly teach and train women in making decisions; increasing their self-confidence; and making positive self-concepts about themselves and involving women in the decision-making process. The findings of this study also indicate the need for further research investigating the role of cultural factors in explaining such a phenomenon, why women stay in poor marital relationships, and how to empower women who are living with abuse, gender preference and forced relationships with an impact on family.

### Limitation

This is a small sample size to describe sensitive cultural issues and experiences regarding gender preference, marital relationship or wife beating among women applicable to women living in these mining communities in Goa. The findings can be transferred to similar populations and settings in the mining community in India.

### Appendix A. Semi-structured in-depth interview guide

1. How would you describe your work?

2. Describe any event of severe or critical illness in your life and how you were cared for?

3. Did you experience anything special or different about problems related to pregnancy, childbirth, after childbirth, childlessness? During your pregnancy, delivery or postpartum period have did you receive the following care or carried out any of the following activities?

4. During the last twelve months, have you been harmed or threatened by your husband (e.g. abused, hit, slapped, kicked, beaten, pinched, pushed, shoved, punched, pulled hair, threatened with words, weapon, mistreated, assaulted, or robbed)? Have you suffered any injury due to your husband’s violent/ aggressive behaviour?

5. Are there any fights/ quarrels/ heated arguments in your home over any issues? How do the consequences of these fights affect you or your family?

6. What type of injury or risks did you face at work in the past? In your experience at your work place, what are the problems you face (e.g. alcoholism…)?

7. During the last twelve months, have you been harmed or threatened by any other person at home, workplace, neighborhood or community (e.g. abused, hit, slapped, kicked, beaten, pinched, pushed, shoved, punched, pulled hair, threatened with words, weapon, mistreated, assaulted, or robbed)? Are there any fights/ quarrels/ heated arguments in your neighborhood or community over any issues? How do the consequences of these fights affect you or your family?

8. Describe any of the salient moments or experiences in your life that were critical in shaping your present positions or conditions at your natal or marital home.

9. As a homemaker, what are the problems that you face (eg. wife-beating, alcoholism, harassment, many children in few years, walk long distances for firewood and water, long hours of work, scarcity of health care facilities, limited access to information, lack ownership of assets, no place for sharing problems)?

10. How do you participate with your husband in decision-making processes at home?

11. What is your present or future apprehensions/ commitments/ plans (personal, family, children, home)? What opportunities, resources and responsibilities do you have in your family (e.g. education, decisions, household work, food consumption, facilities)?

12. How do you think that your life has been good, satisfying or content? Does this change at different times of her life? If so, how does this change?

13. How do you support your husband in decision making at home? How does he react when you disagree to his decisions?

14. In the event of conflict with any family member, how does your husband support you? How often do you and your husband opinions match on family matters or decisions?

15. How do you take care of each other views or concerns in matters relating to daily events or family?

## Abbreviations

RH: Reproductive health

## Competing interests

The authors declare that they have no potential competing interests.

## Authors’ contributions

All authors meet the criteria for authorship, have designed, provided analysis and interpreted the data, drafted, revised and approved the final article and those entitled to authorship are listed as authors. MSD and KSN conceived of the study conception and design, data collection/ acquisition, and provided analysis and interpretation. MSD, KSN, GS, and RV drafted the first manuscript, provided critical revision of manuscript for important intellectual content and provided final approval of version to be submitted. All authors read and approved the final manuscript.
